# Extracting principal diagnosis, co-morbidity and smoking status for asthma research: evaluation of a natural language processing system

**DOI:** 10.1186/1472-6947-6-30

**Published:** 2006-07-26

**Authors:** Qing T Zeng, Sergey Goryachev, Scott Weiss, Margarita Sordo, Shawn N Murphy, Ross Lazarus

**Affiliations:** 1 Decision Systems Group, Brigham and Women's Hospital, Boston, MA, USA; 2 Channing Laboratory, Brigham and Women's Hospital, Boston, MA, USA; 3 Laboratory of Computer Science, Massachusetts General Hospital, Harvard Medical School, Boston, MA, USA

## Abstract

**Background:**

The text descriptions in electronic medical records are a rich source of information. We have developed a Health Information Text Extraction (HITEx) tool and used it to extract key findings for a research study on airways disease.

**Methods:**

The principal diagnosis, co-morbidity and smoking status extracted by HITEx from a set of 150 discharge summaries were compared to an expert-generated gold standard.

**Results:**

The accuracy of HITEx was 82% for principal diagnosis, 87% for co-morbidity, and 90% for smoking status extraction, when cases labeled "Insufficient Data" by the gold standard were excluded.

**Conclusion:**

We consider the results promising, given the complexity of the discharge summaries and the extraction tasks.

## Background

Clinical records contain much potentially useful information in free text form. In electronic medical records (EMR), information such as family history, signs and symptoms and personal history of drinking and smoking are typically embedded in text descriptions provided by clinicians in the form of progress notes and in more formalized discharge summaries. Even when coded data are available (e.g. billing codes for principal diagnoses and co-morbidities), they may not always be accurately assigned or widely utilized and may be subtly influenced by financial incentives.

Natural language processing (NLP) techniques have demonstrated the potential to unlock such information from text. The MEDLEE system developed by Friedman et al [1], the Symtxt system developed by Haug [2], the statistical NLP tool by Taira et al [3], and the widely used MetaMap/MMtx [4] by National Library of Medicine researchers are examples of medical NLP systems. These and other systems have been evaluated for a variety of tasks from extracting pathology findings to identify pneumonia cases [5-12].

One challenge with many previously described medical NLP tools is that they are not easy to adapt, generalize and reuse. There had have been few examples of a NLP system developed by one institution adapted for use by an unrelated institution. One reason is that medical NLP programs are often tailored to domain or institution-specific document formats and other text characteristics. The intellectual property of software, of course, has also been an obstacle in sharing.

As a part of the National Center for Biomedical Computing I2B2 (Informatics for Integrating Biology & the Bedside) project [13], we have been developing a NLP tool that we refer to as the Health Information Text Extraction (HITEx) tool. Following the example of GATE (General Architecture for Text Engineering) [14] and using GATE as a platform, a suite of open-source NLP modules were adapted or created. We then assembled these modules into pipelines for different tasks.

One of the component projects under I2B2 is a study investigating factors contributing to asthma exacerbation and hospitalization. HITEx was used to extract from discharge summaries the principal diagnoses and co-morbidities associated with a hospitalization, and the smoking status of the patient, from discharge summaries and longitudinal medical record's free form text notes [15].

To evaluate the accuracy of information extracted by HITEx, an asthma expert reviewed 150 discharge summaries of patients with known history of asthma or Chronic Obstructive Pulmonary Disease (COPD) and extracted principal diagnoses, co-morbidities and smoking status information from each report. Since a human's ability to maintain focus tends to decline after hours of review, so the human expert's extraction were confirmed by four other physician members of the I2B2 team. The HITEx results were then compared to the human extractions.

### NLP

HITEx uses GATE as the development platform. GATE is an open-source natural language processing framework; it includes a set of NLP modules, collectively known as CREOLE: a Collection of REusable Objects for Language Engineering [14]. CREOLE contains NLP modules that perform some common tasks, such as tokenizing, part-of-speech (POS) tagging, and noun phrases parsing. The GATE framework can be viewed as a backplane for plugging in CREOLE components. The framework provides various services to the components, such as component discovery, bootstrapping, loading and reloading, management and visualization of data structures, and data storage and process execution. GATE is an active project at the University of Sheffield, UK with a large user community worldwide.

**Figure 1:**
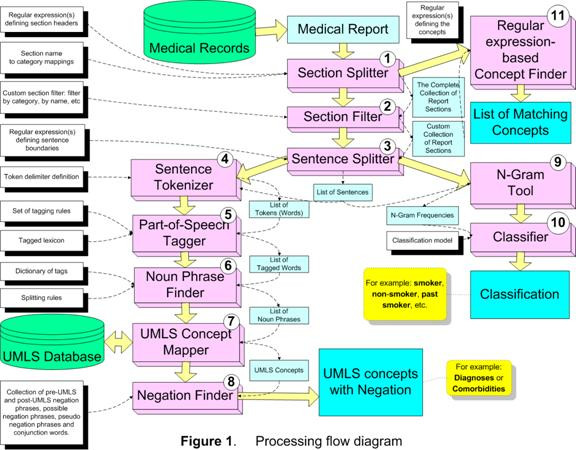
Processing Flow Diagram.

HITEx uses 11 GATE modules (components), two of which were adapted from the CREOLE, and rest were developed specifically for HITEx (see Figure 1):

#### 1. Section splitter

splits the medical report into the sections; assigns the section to the category (categories), which are based on the section headers for each type of the medical document. For the parsing of discharge summaries and outpatient notes, we collected and categorized over 1000 section headers. For example, "principal diagnosis" is categorized as "Primary Diagnosis", while the "discharge medications" header is mapped to both the Discharge and Medications categories. The header collections are not part of the section splitter, but supplied to the splitter as a configuration file.

#### 2. Section filter

selects the subset of sections based on the selection criteria, such as category name, section name, etc. This module uses a simple expression language that allows rather complex criteria expressions to be created.

#### 3. Sentence splitter

splits the section into sentences. The module relies on the set of regular expression-based rules that define sentence breaks.

#### 4. Sentence tokenizer

splits the sentence into the tokens (words). The module uses the extensive set of regular expressions that define both token delimiters and special cases when certain punctuation symbols should not be used as token delimiters (e.g. decimal point in numbers, period in some multi-word abbreviations, etc).

#### 5. POS tagger

assigns part-of-speech tags to each word (token) in the sentence. This module is based on the Brill-style, rule-based POS tagger, originally written by Mark Hepple [16] as a plug-in for the Gate framework.

#### 6. Noun phrase finder

groups POS-tagged words into the noun phrases using the set of rules and the lexicon. This module is an implementation of the Ramshaw and Marcus transformational learning-based noun phrase chunker [17]. The original version is available as a Gate framework plug-in.

#### 7. UMLS concept mapper

maps the strings of text to UMLS (Unified Medical Language System) concepts. The module first attempts exact match; when exact matches are not found, it stems, normalizes and truncates the string. For instance, "failures of heart" is mapped to the concept "Heart Failure" and "back pain and asthma" is mapped to concepts "Back Pain" and "Asthma".

#### 8. Negation finder

assigns the negation modifier to the existing UMLS concepts. Currently, this module is an implementation of NexEx-2 negation algorithm developed by Chapman et al. [18].

#### 9. N-gram tool

extract n-word text fragments along with their frequency from a collection of text.

#### 10. Classifier

takes a smoking-related sentence to determine the smoking status of a patient. The classifier is a support vector machine (SVM) using single words as features. It was trained and tested on a data set of about 8500 smoking-related sentences through 10-fold cross-validation. To create the classifier, we experimented with naïve Bayes, SVM and decision trees, and 1–3 word phrase features using Weka [19], which is a publicly available tool kit. The detailed description of the experiments can be found in a previous publication [15].

#### 11. Regular expression-based concept finder

finds all occurrences of the concepts defined as a regular expression in the input chunk of text. For example: medications, smoking keywords, etc.

Each module expects a set of parameters/configuration files. Take the task of extracting principal diagnosis for example, we would specify the primary diagnosis headers for the Section Filter module. For each task, a different pipeline of modules may be assembled. To extract diagnoses, Section Splitter, Section Filter, Sentence Splitter, Sentence Tokenizer, Part-of-Speech, Noun Phrase Finder, UMLS Concept Mapper and Negation Finder modules would be applied sequentially. While for extracting smoking status, Section Splitter, Section Filter, Sentence Splitter, N-gram tool and Classifier are formed into a pipeline.

Except the smoking classifier module, none of the modules had been formally evaluated. In ad-hoc testing, results obtained from the modules were satisfactory.

### Asthma and COPD

Asthma is the most common disease of the airways in children and adults [20]. Although most asthma patients are generally well controlled on modern medications, acute asthma flare-ups happen in a small subset of patients, occasionally leading to a life-threatening "status asthmaticus" exacerbation episode, often associated with hospitalization. Identifying preventable risk factors may allow clinicians to decrease these unfortunate events. In order to perform appropriate statistical analyses, measures of important environmental exposures are needed because these are known to substantially increase exacerbation risk, including co-morbidity, medication use, tobacco smoking history and smoking status. Since these were not available in coded form in the large data collection we are using, we sought to obtain estimates for these covariates from the case notes and discharge summary text corpus available for each patient, using NLP tools.

While diagnosis is often the first item a medical NLP application extracts, distinguishing principal diagnosis from co-morbidities in text is a much more subtle task, because it requires us to reliably differentiate present from historical, and primary from secondary. In terms of complexity, inferring smoking status from text similarly requires distinguishing present from historical, and often requires subtle distinctions between syntactically similar textual expressions such as "recent non-smoker" and "never smoked".

For billing purpose, professional coders (typically not clinicians) assign ICD9 codes to each encounter such as a hospitalization. While these codes are very useful, a number of studies have shown that they are not very reliable records of diagnoses [21-23]. Smoking status, on the other hand, is often not encoded.

## Methods

A large data set containing records on approximately 97,000 asthma and COPD patients was obtained from the Partners' Health Care Research Patient Data Repository (RPDR). The RPDR data warehouse includes a wide variety of records (not deidentified) including free text, administrative codes, laboratory codes and text, and numerous other data sources for all encounters for all patients at all Partners facilities. The patients included in our data set had one or more asthma or COPD related admission diagnosis (determined by their ICD9 billing codes) in one or more of their RPDR records. The RPDR population includes 90–98% of the total Partners patient population. The study was approved by the Brigham and Women's Hospital's institutional review board; the protocol number is 2004P002260 (Subphenotypes in Common Airways Disorders).

In the evaluation, we focused on discharge summaries, in particular, those related to hospitalization caused by asthma or COPD exacerbation. We collected a random sub-sample of 150 discharge summaries from the data set that either has had an asthma or COPD related ICD9 billing code or contains an asthma or COPD related string from an extensive list of related concepts and names that we manually identified.

An asthma expert (STW) reviewed the 150 reports and answered the following questions for each report:

1. Principal diagnosis include asthma: yes/no/insufficient data

2. Principal diagnosis include COPD: yes/no/insufficient data

3. Co-morbidities include asthma: yes/no/insufficient data

4. Co-morbidities include COPD: yes/no/insufficient data

5. Smoking status: current smoker, past smoker, non smoker, patient denies smoking, insufficient data

Similarly, HITEx was used to answer the same 5 questions for each report. First, we created three HITEx pipelines to extract principal diagnosis, co-morbidities and smoking status, respectively. The principal diagnosis and co-morbidities were then processed to determine if they contained an asthma or COPD diagnosis. To do so, we used the relationship table (MRREL) in the UMLS. All descendants of the asthma and COPD concepts were considered to be a type of asthma and COPD. Diagnoses extracted from the principal diagnosis sections (determined by the section headers) were deemed as principal. When we could not determine if a diagnosis was primary or secondary because of the lack of header information, it was considered to be primary by default.

We also used ICD9 codes to answer the questions regarding the principal diagnosis and co-morbidities. The ICD9 hierarchy was used to determine if a code is asthma or COPD related. The codes are: Asthma – 493.*; COPD – 490–492.*, 494–496.*, 466.*.

For asthma/COPD principal diagnosis and co-morbidities, we compared the HITEx and ICD9 answers to that of the human expert. For smoking status, HITEx results were compared to the human expert's.

Generally speaking, NLP programs infer "yes" for a diagnosis if certain string patterns are found, "no" if they are not found, but often do not have a notion of "insufficient data". On the other hand, the human label of "insufficient data" could result from the absence of explicit information, the presence of ambiguous information or the presence of conflicting information. To compare HITEx results to the human ratings, we treated the "insufficient data" label in three ways: exclude cases with the label, regard it as "yes", and regard it as "no". In the case of smoking, though, non-smoker status are often explicitly stated so "insufficient data" was interpreted to mean that no smoking-related information was found.

In addition, we experimented with two ways to combine ICD9 and HITEx results to improve the diagnosis extraction performance:

NLP and ICD9:

- Both are 'YES' → 'YES'

- everything else → 'NO'

NLP or ICD9:

- either one is 'YES' → 'YES'

- everything else → 'NO'

We calculated the accuracy of HITEx, ICD9 and HITEx-ICD9 combinations, i.e. the percentage of cases which HITEx, ICD9 or HITEx-ICD9 combinations agreed with the expert. Sensitivity and specificity were also calculated, assuming the human expert as the correct classifier. In practice, a human's ability to maintain focus tends to decline after a few hours of this relatively tedious chart review, so the human expert's answers were confirmed by four other physician members of the I2B2 team. Some obvious errors and omissions were corrected by a consensus of clinicians at the team meeting.

## Results

Comparing to the expert, the accuracy of HITEx for principal diagnosis extraction was 73% to 82% and for co-morbidity was 78% to 87% depending on how the expert label "insufficient data" was treated. The HITEx accuracy was higher than ICD9 in every category, though sometimes only by a small margin (Table 1).

**Table 1:** The accuracy of ICD9, HITEx and the combinations of ICD9 and HITEx for principal diagnosis and co-morbidity extraction.

		**ICD9**	**HITEx**	**ICD9 and HITEx**	**ICD9 or HITEx**
**Exclude Insufficient Data**	Principal	80.08%	81.64%	75.78%	85.94%
	Co-morbidity	82.57%	86.72%	88.80%	80.50%
**Insufficient Data = Yes**	Principal	71.57%	73.20%	66.34%	78.43%
	Co-morbidity	74.18%	77.45%	79.41%	72.22%
**Insufficient Data = No**	Principal	78.76%	79.74%	76.80%	81.70%
	Co-morbidity	83.33%	84.64%	90.52%	77.45%

The combination of HITEx or ICD9 resulted in better accuracy of principal diagnosis extraction – 78% to 86%, while the combination of HITEx and ICD9 resulted in better accuracy of co-morbidities extraction – 79% to 91%.

HITEx achieved 64% to 77% sensitivity and 82% to 87% specificity in principal diagnosis extraction; and 40% to 71% sensitivity and 87% to 89% specificity in co-morbidity extraction (Table 2). Precision is reported in Table 3. We believe the lower sensitivity and higher specificity of co-morbidity extraction was a result of HITEx treating a diagnosis as primary when we could differentiate primary from secondary. Overall, HITEx had better sensitivity and worse specificity than ICD9 (60% to 72% sensitivity and 85% to 91% specificity in principal diagnosis extraction; and 11% to 15% sensitivity and 90%–91% specificity in co-morbidity extraction).

**Table 2:** The accuracy of ICD9, HITEx and the combinations of ICD9 and HITEx for principal diagnosis and co-morbidity extraction.

		**ICD9 (Sens/Spec)**	**HITEx (Sens/Spec)**	**ICD9 and HITEx (Sens/Spec)**	**ICD9 or HITEx (Sens/Spec)**
**Exclude Insufficient Data**	Principal	72.52%/90.91%	76.69%/86.99%	56.39%/96.75%	92.42%/80.99%
	Co-morbidity	14.81%/91.12%	70.37%/88.79%	11.11%/98.60%	74.07%/81.31%
**Insufficient Data = Yes**	Principal	60.22%/90.91%	63.93%/86.99%	45.90%/96.75%	78.02%/80.99%
	Co-morbidity	11.11%/90.53%	39.68%/87.24%	04.76%/98.77%	46.03%/37.86%
**Insufficient Data = No**	Principal	72.52%/85.38%	76.69%/82.08%	56.39%/92.49%	92.42%/74.85%
	Co-morbidity	11.11%/90.32%	66.67%/86.38%	07.41%/98.57%	70.37%/78.14%

**Table 3:** The precision of ICD9 and HITEx for principal diagnosis and co-morbidity extraction.

		**ICD9**	**HITEx**	**ICD9 and HITEx**	**ICD9 or HITEx**
**Exclude Insufficient Data**	Principal	82.32%	82.28%	81.06%	87.41%
	Co-morbidity	53.46%	69.71%	69.88%	64.63%
**Insufficient Data = Yes**	Principal	75.64%	75.03%/	75.01%	78.24%
	Co-morbidity	50.99%	64.51%	65.00%	60.63%
**Insufficient Data = No**	Principal	79.69%	79.35%	79.22%	83.19%
	Co-morbidity	50.53%	64.11%	62.48%	60.14%

The combination of HITEx or ICD9 led to better sensitivities – 78% to 92% for principal diagnosis extraction and 46% to 74% for co-morbidities extraction, while the combination of HITEx and ICD9 resulted in better specificities – 93% to 97% for principal diagnosis extraction and 99% for co-morbidities extraction.

The accuracy of smoking status extraction was 90%. The sensitivities and specificities range from 60% to 100% and 93 to 99% respectively (Table 3). Precision is reported in Table 5.

**Table 4:** The sensitivity and specificity of HITEx for smoking status extraction.

	**Current Smoker**	**Never Smoked**	**Denies Smoking**	**Past Smoker**	**Not mentioned/Insufficient Data**
Sensitivity	90.32%	60.00%	100.00%	77.78%	94.57%
Specificity	92.56%	98.60%	99.34%	99.26%	95.08%

**Table 5:** The precision of HITEx for smoking status extraction.

	**Current Smoker**	**Never Smoked**	**Denies Smoking**	**Past Smoker**	**Not Mentioned/Insufficient Data**	**Average**
Precision	75.68%	75.00%	66.67%	93.33%	96.67%	81.47%

## Discussion

This study evaluated the information extraction accuracy of a new, portable NLP system HITEx, by comparing it to an expert human gold standard. When "Insufficient Data" cases were excluded, the accuracy of HITEx for principal diagnosis extraction was 82% and for co-morbidities was 87%. The sensitivity and specificity of HITEx were 77% and 87% for principal diagnosis and 70% and 89% for co-morbidity extraction. The accuracy of smoking status extraction was 90% and the sensitivities and specificities range from 60% to 100% and 93 to 99% respectively.

Since ICD9 codes are generally available, we found that it could be used to complement the HITEx results: the combination of HITEx or ICD9 improves the accuracy to 86% for principal diagnosis, while the combination of HITEx and ICD9 improves the accuracy to 89% for co-morbidities. The combination of HITEx or ICD9 led to better sensitivities – 92% for principal diagnosis and 74% for co-morbidity, while the combination of HITEx and ICD9 resulted in better specificities – 97% for principal diagnosis and 99% for co-morbidity.

The HITEx performance we report here is comparable to the results from a number of previous studies in the literature [1,24,25], though there had also been better sensitivity and specificity reported for certain NLP applications [26,27]. We find the HITEx results promising for the following reasons:

1. The discharge summaries we processed are a far "messier" corpus than narrower domain (e.g. radiology or pathology) reports reported in many previous studies. For example, each individual unit in each individual hospital within the Partners system tends to have its own specific style for these summary documents, with numerous broadly common features but also many idiosyncratic, local conventions.

2. The tasks we undertook in this study were relatively challenging. We are not aware of prior NLP attempts to differentiate principal diagnoses and co-morbidities. Determination of smoking status is more complicated than extracting the status of fever or headache, because smoking status is itself a relatively complex construct, and unfortunately is rarely the focus of specific attention in the discharge summary texts we encountered.

3. We made a decision not to embed decision making logic in the NLP system: for example, inferring HIV status from AZT or inferring pneumonia from infiltrate. While such logic is very useful, we believe it should be developed and evaluated separately.

HITEx modules 3 through 7 provide the same functionality available in the MetaMap/MMTx. Instead of using MetaMap/MMTx, we adopted or developed these modules to allow local manipulation of the UMLS tables (e.g. add and remove synonyms) as well as utilization of the sentence and POS tags in other modules. Though no formal evaluation has been done, we have been collaborating with the MMTx's developer (Mr. Divita) and have observed that the HITEx concept mapping capabilities were similar to that of the MMTx.

When we analyzed the disagreement between the human gold standard and the NLP program, we found that while HITEx had made some mistakes, the disagreements in a large number of cases were a result of the human expert's extensive domain knowledge. Here are some examples: In one case, COPD was listed as one of the final diagnoses and asthma was not, but the expert chose asthma not COPD as the principal diagnosis. In another case, though the text did not mention anything about smoking, the expert inferred the non-smoking status from the patient's age (5 years old). When the designation of primary and secondary diagnoses not made explicit in the text, the expert could still differentiate them or give label "insufficient data". Our NLP program does not have this level of sophistication.

Of course, the expert human is not infallible either. In this study, we first used one domain expert as the basis for the gold standard. Because extracting information from text is a tedious task, it was necessary for us to correct some obvious oversights in a second pass.

This study over-sampled asthma and COPD cases, which biased the evaluation. This was partially necessary because the prevalence of asthma and COPD in patients was relative low and the prevalence of asthma and COPD related hospitalization was even lower. When we manually reviewed the principal diagnoses of hospitalization in those patients who had at least one asthma- or COPD-related billing code in the past 10 years, we found most of them not caused by asthma or COPD exacerbations. A very large number of hospitalizations appeared to be associated with elderly patients with other serious diseases (e.g. cancer and heart disease).

We mainly depended on one expert in the study, though a few other researchers later participated in the review of the gold standard; ideally, at least 3 experts working independently would be desirable. This evaluation gives an estimate of the HITEx extracted data quality for the airways disease project. More rigorously designed evaluations of HITEx are being planned for the future.

One issue that deserves further thought is how to establish realistic and reliable gold standards. First, there is inherit ambiguity in text and clinical conditions which need to be accounted for in the gold standard (e.g. differentiate "insufficient data" from "no data"). Second, a domain expert may need to work with lay reviewers to come up with the gold standard for NLP. Domain experts are sometimes influenced by their clinical experience (e.g. most COPD patients to be past or current smoker), while lay reviewers tend to rely on text alone. Although eventually we want to have a computer system to behave like a human expert, NLP is a different task from clinical decision making. We should probably not try to build expert knowledge such as "bipolar disorder implies smoker" into text processing applications – these rules may be useful but ideally might be applied in a separate processing step.

Finally, in terms of smoking history and status, the generally accepted epidemiological "gold standard" is patient self-report using a structured and standardized questionnaire. From a practical point of view, it seems difficult to imagine a more reliable gold standard measure than self-report to use in evaluating NLP tools, since most of the text corpora available to us in medical records are created by a health care provider after talking to the patient. Unfortunately, there is a rich literature on recall bias and other problems with this approach, suggesting that this method is not always free from error [28,29]. Without wishing to engage in debates about what truth really is, it is clear that the task of evaluating any NLP system is made more vexing by the complex layers between the contents of the corpora available and the historical facts as related to a health care provider by a patient.

The evaluation helped us identify a few important development areas for HITEx:

1. Accurate identification of negation and uncertainty modifiers: HITEx has a negation finder module that uses the Chapman algorithm [18], the error rate of which is between 5% to 10%.

2. Differentiation between family and personal history: not all diagnosis mentioned referred to the patients.

3. Extraction of temporal modifiers: this is particularly important for interpreting the smoking status correctly.

We also recognize the need to create certain post-processing functionalities of HITEx results to support asthma and other research. Taking principal diagnoses extraction for instance, we may train a classifier to perform this task based occurrence frequency when diagnoses are not explicitly labeled as primary or secondary.

## Conclusion

Free text clinical records contain a large amount of useful information, and NLP has the potential to unlock this wealth of information. One challenge with medical NLP tools is that they are not easy to adapt, generalize and reuse. We have developed an open source, reusable, component-based NLP system called HITEx. HITEx was evaluated on 150 discharge summaries and the results were compared to a human-created gold standard.

The evaluation showed the accuracy of HITEx in principal diagnosis, co-morbidity and smoking status extraction to be in the 70% to 90% range, generally comparable to a number of previously reported NLP systems. HITEx performed slightly better than ICD9 in diagnosis extraction. Combining HITEx and ICD9 could further improve the accuracy, sensitivity and specificity. We found these results encouraging given the complexity of the tasks, and heterogeneity of the discharge summaries.

## Competing interests

The author(s) declare that they have no competing interests.

## Authors' contributions

QTZ designed the HITEx system and drafted the manuscript; SG was the main developer of HITEx; SW provided expert review of the discharge summaries; MS was responsible for the smoking status classification module in HITEx; SNM collected the discharge summaries and participated in the evaluation study design; RL conceived the evaluation and helped to draft the manuscript. All authors have read and approved the final manuscript.

## Pre-publication history

The pre-publication history for this paper can be accessed here:


